# Downward terrestrial gamma-ray flash associated with collision of lightning leaders

**DOI:** 10.1126/sciadv.ads6906

**Published:** 2025-05-21

**Authors:** Yuuki Wada, Takeshi Morimoto, Ting Wu, Daohong Wang, Hiroshi Kikuchi, Yoshitaka Nakamura, Eiichi Yoshikawa, Tomoo Ushio, Harufumi Tsuchiya

**Affiliations:** ^1^ Graduate School of Engineering, The University of Osaka, 2-1 Yamadaoka, Suita, 565-0871 Osaka, Japan.; ^2^ Faculty of Science and Engineering, Kindai University, 3-4-1 Kowakae, Higashiosaka, 577-8502 Osaka, Japan.; ^3^ Department of Electrical, Electronic and Computer Engineering, Gifu University, 1-1 Yanagido, Gifu, 501-1193 Gifu, Japan.; ^4^ Center for Space Science and Radio Engineering (SSRE), The University Electro-Communications, 1-5-1 Chofugaoka, Chofu, 182-8585 Tokyo, Japan.; ^5^ Department of Electrical Engineering, Kobe City College of Technology, 8-3, Gakuen-Higashimachi, Nishi-ku, Kobe, 651-2194 Hyogo, Japan.; ^6^ Electrical and Computer Engineering, Colorado State University, Fort Collins, CO 80523-1373, USA.; ^7^ Aeronautical Technology Directorate, Japan Aerospace Exploration Agency, 6-13-1 Osawa, Mitaka, 181-0015 Tokyo, Japan.; ^8^ Nuclear Science and Engineering Center, Japan Atomic Energy Agency, 2-4 Shirakata, Tokai-mura, 319-1195 Ibaraki, Japan.

## Abstract

Lightning discharges can produce transient gamma-ray emissions called terrestrial gamma-ray flashes (TGFs), which originates from electrons accelerated to relativistic energies in strong electric fields. However, it is not yet understood how lightning produces an enormous number of relativistic electrons in dense atmospheres. We present that, thanks to a ground-based observation with optical, radio frequency and high-energy measurements focused on television transmission towers in Kanazawa, Japan, a TGF and a cloud-to-ground discharge of −56 kA occurred when a downward negative leader was colliding with an upward positive leader from the tower. Because the cloud-to-ground current followed the TGF by 30 μs, the TGF started when two leaders approached each other. Our results indicate that an immense number of electrons were produced and accelerated to relativistic energies in a strong and compact electric-field region between the two leaders.

## Teaser

An intense gamma-ray flash occurred when two lightning paths were extended from a thundercloud and the ground and were colliding.

## INTRODUCTION

Terrestrial gamma-ray flashes (TGFs) are submillisecond bursts of high-energy photons, generally coincident with lightning discharges. After the serendipitous discovery by the Burst And Transient Source Experiment onboard the Compton Gamma Ray Observatory (*1*), they have been detected mainly by space-borne detectors such as the Reuven Ramaty High Energy Solar Spectroscopic Imager (*2*), the Astro-rivelatore Gamma a Immagini LEggero (*3*), Fermi (*4*), and the Atmosphere-Space Interactions Monitor (*5*). TGFs typically have an energy spectrum of gamma-ray photons with a continuum component up to >10 MeV (*2*) and last for tens to a few hundreds of microseconds (*5*, *6*). While space missions have detected a vast majority of TGFs, a limited number of TGFs have also been detected by airborne (*7*, *8*) and ground-based detetors (*9*–*15*).

TGFs are thought to originate from bremsstrahlung photons of relativistic electrons accelerated by lightning in the dense atmosphere. The relativistic runaway electron avalanche (RREA) is a widely accepted theory to produce and multiply relativistic electrons by strong electric fields in the atmosphere (*16*). On the other hand, previous studies have pointed out that the RREA scheme is not enough to explain the number of relativistic electrons involved in a TGF, typically 10^16^ to 10^19^ (*17*, *18*). While the relativistic feedback (*19*, *20*) and the thermal runaway (*21*, *22*) processes have been proposed, the source mechanism of TGFs is not yet understood.

Multifrequency observations of TGFs are becoming more important recently because parent lightning flashes can be in detail observed in radio frequency (RF) (*10*, *13*, *15*, *23*–*27*) and optical (*5*, *10*, *14*) bands. In particular, RF observations have been used to identify lightning processes associated with TGFs. A subset of TGFs is known to be associated with a specific class of large-amplitude RF pulses (*13*, *15*, *26*, *28*–*30*), and these large-amplitude pulses are thought to be always accompanied by TGFs (*28*, *31*).

In the Hokuriku region of Japan, TGFs with downward gamma-ray beams called downward TGFs have been detected on the ground, with a similar number of relativistic electrons to TGFs seen from the space (*12*, *13*). Because of its unique characteristics, winter thunderstorms along the coast of the Sea of Japan in the Hokuriku region have been investigated since the 1970s (*32*–*34*). Since winter thunderstorms have a lower charge-center feature due to the lower temperature than summer ones, TGFs tend to occur near the ground, and gamma rays can reach the ground without being much attenuated in the atmosphere. It facilitates detections of downward TGFs at sea level.

We have conducted an observation campaign of downward TGFs with high energy, RF, and optical detectors, aiming at winter thunderstorms in Japan. Here, we report a multifrequency observation of a downward TGF associated with the collision of two leaders in opposite polarities and directions. We discuss a possible scenario to produce the downward TGFs.

## RESULTS

### TGF observation

**Fig. 1.**
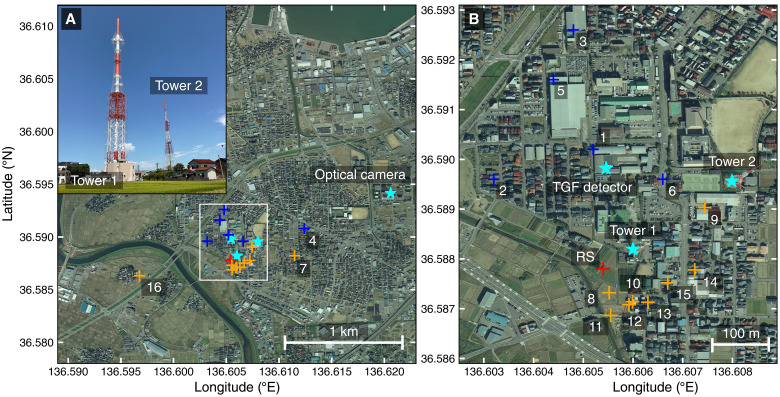
Maps of the observation site in Kanazawa. (**A**) Overview of the observation site and a photo of two television transmission towers. (**B**) Expanded view of the left panel. The area of the right panel is indicated by the white frame on the left panel. Four cyan stars show the positions of the two television transmission towers, the TGF detector, and the optical camera. Blue and orange cross markers indicate the source position of RF pulses located by the LF network and DALMA, respectively. The numbers beside the blue and orange markers correspond to those in the bottom panels in Fig. 2. The red cross shows the source position of the large LF pulse associated with the TGF, located by the LF network. The background aerial photos are provided by the Geospatial Information Authority of Japan. The photo of the towers was taken by Yuuki Wada, Osaka University.

We conducted a multi-wavelength observation of downward TGFs, targeting two adjacent television transmission towers that are located in Kanazawa, Ishikawa Prefecture, Japan, and are frequently struck by lightning in winter. Figure 1 shows the location of the two towers and detectors. Tower 1 and Tower 2 are located at 36.588°N, 136.606°E, and 36.590°N, 136.608°E, respectively. A detector specialized for observing TGFs (hereafter TGF detector) was installed at Ishikawa Prefectural Kanazawa College of Industry and Technology near the towers.

The TGF detector is divided into radiation and RF detection sections. The radiation detection section is equipped with two sizes of plastic scintillators: 1 cm by 1 cm by 1 cm and 5 cm by 5 cm by 5 cm. The former is the low-sensitivity channel, and the latter is the high-sensitivity channel. They are connected to a Hamamatsu R7600U photomultiplier tube, and the output signals are amplified and waveform-shaped by analog circuits. The time constant of the waveform shaping is 50 ns for the low-sensitivity channel and 100 ns for the high-sensitivity one. A voltage of −520 V is applied to the photomultiplier tubes. The radiation detection section is entirely enclosed in a metal case to protect it from electromagnetic noise. Energy calibration was performed using 662-keV gamma rays from a ^137^Cs source and a Compton edge with the plastic scintillators. The energy range is up to 2.5 MeV for the low-sensitivity channel and up to 5.0 MeV for the high-sensitivity channel. The RF detection section consists of a flat-plate antenna and a bandpass filter. The broadband RF signal received by the antenna is filtered to the narrow very-high-frequency (VHF) band of 28 to 32 MHz by the bandpass filter.

Waveform signals from the radiation and RF detection sections are recorded with a PicoScope 6402D, which is a USB oscilloscope that can record four channels simultaneously. The vertical and horizontal resolutions are 8 bits and 3.2 ns/312.5 Msps (mega samples per second), respectively, and 9.6 ms before the trigger and 96.0 ms after the trigger are recorded in memory. To acquire waveforms only when lightning occurs, the VHF signals are used as the trigger source. This prevents triggers from constantly arriving environmental radiation and records only the necessary data. This system is time-calibrated with a time-synchronization signal generated from the pulse-per-second signal of the Global Navigation Satellite System (GNSS). We used a NEO-6M–based GNSS module with an average timing accuracy of 30 ns and a field programmable gate array (FPGA)–based clock generator with an accuracy of 100 ns. Therefore, the absolute timing accuracy of the TGF detector when the GNSS signal is received is better than 200 ns.

### RF observation

**Fig. 2.**
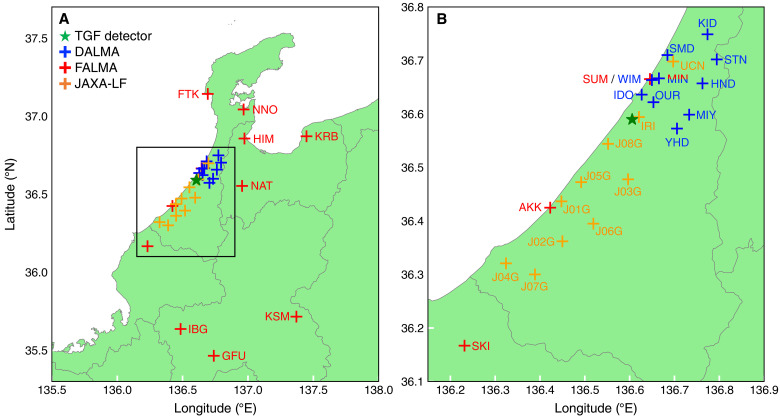
Maps of TGF detector, LF network, and DALMA. (**A**) Entire view. (**B**) Expanded view. Its area is indicated by the black frame in (A). The green star maker shows the position of the TGF detectors. The red, orange, and blue cross markers show the positions of FALMA, JAXA-LF, and DALMA antennas. The station names are listed beside the markers. The MIN site has both FALMA and DALMA antennas. The LF network consists of FALMA and JAXA-LF antennas. The green region indicates lands, and the gray lines show the coastline and the borders of prefectures.

RF emissions from lightning are monitored in two different bands. One is the broadband low-frequency (LF) band. This observation used the fast antenna lightning mapping array (FALMA) operated by Gifu University (*35*) and the broadband LF network (JAXA-LF) operated by JAXA and Kindai University. Each FALMA station has a flat antenna, and the received signal passes through a 0.5- to 500-kHz bandpass filter and then is digitally sampled at 16 bits and 1 Msps. Each JAXA-LF station has a flat antenna similar to FALMA, but after passing through a 2-MHz low-pass filter, the received signal is digitally sampled at 16 bits and 5 Msps. To perform combined analysis with FALMA, a 0.5- to 500-kHz digital bandpass filter is applied to the digitally sampled data of JAXA-LF. Hereafter, the two systems are collectively referred to as the LF network. The operating stations of the LF network as of 30 January 2023 are also shown in Fig. 2.

The localization of RF sources using the LF network was performed with the time-of-arrival method. Because of the long wavelength of the LF band, the positioning accuracy in the altitude direction is relatively low compared to the horizontal direction. The IRI station of JAXA-LF is installed at the Industrial Research Institute of Ishikawa near the towers (1.5 km from Tower 1), and it is possible to improve the locating accuracy in the altitude direction if LF pulses are properly recorded by this station. Other LF pulses not recorded by the IRI station are two-dimensionally located. The absolute timing of each LF network station is calibrated by GNSS signals with an accuracy of 50 ns, and typical location accuracy around the towers is 300 m.

The other band is the medium-frequency to high-frequency (MF-HF), and we used the discone antenna lightning mapping array (DALMA) operated by Gifu University (*36*). DALMA consists of 12 stations, whose locations are shown in Fig, 2. Each DALMA station is equipped with a discone antenna and performs 16-bit digital sampling at 25 Msps. The source location of lightning pulses is determined using the time-of-arrival method. Note that amplitude modulation broadcast waves are strongly mixed in the MF band, so steady signals other than lightning discharges are digitally filtered, and the filtered waveform is analyzed. The absolute timing of each DALMA station is calibrated by GNSS signals with an accuracy of 50 ns, and typical location accuracy around the towers for both horizontal and vertical directions is 50 m (*36*, *37*).

### Optical observation

An optical camera system is used to provide visual ground truth of lightning striking the two towers. The camera is installed at the Industrial Research Institute of Ishikawa, 1.5 km from Tower 1 (the same location as the IRI station of the LF network, shown in Fig. 1). We use a Sony mirrorless camera α6400 with a Sony wide-angle lens SEL11F18 and an ND16 filter. The angle of view is 27 mm in 35 mm equivalent. Exposure, shutter speed, and sensitivity are fixed at F1.8, 1/60 s, and ISO100, respectively. The video signal is outputted at 30 fps and processed by a video capture board connected to a computer. The video signals captured in the computer are recorded by the capture software UFOCaptureHD2 only when there are large brightness changes between frames. The timing is synchronized with the network time protocol with an accuracy of ∼1 s. The camera system is enclosed in a waterproof box and takes videos through a transparent acrylic panel of the box.

### Detection of downward TGF

**Fig. 3.**
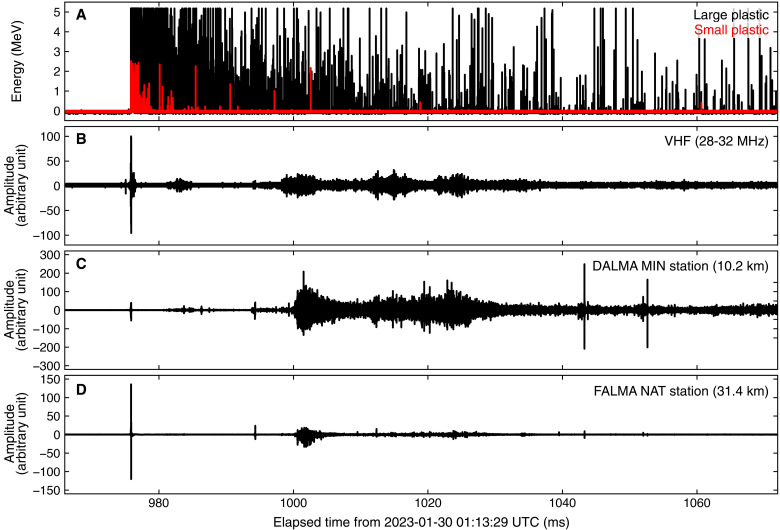
Entire view of radiation and RF waveforms. (**A**) Waveforms of the high-sensitivity/large (black) and low-sensitivity/small (red) plastic scintillators onboard the TGF detector. (**B**) Waveform of the VHF band (28 to 32 MHz) recorded by the TGF detector. (**C**) Waveform of the HF band recorded by the MIN station of DALMA. (**D**) waveform of the LF band recorded by the NAT stations of FALMA. The propagation time in the HF and LF waveforms between the stations and Tower 1 is corrected, while that of the radiation and VHF waveforms (which is estimated to be within a few microseconds) is not corrected.

At 01:13:29 UT on 30 January 2023, the TGF detector recorded a burst of VHF pulses and gamma rays associated with a lightning flash. Figure 3 shows the waveforms of gamma rays, VHF, DALMA, and the LF network. At the beginning of the lightning flash, the RF sensor of the TGF detector, the LF network, and DALMA detected a large-amplitude RF pulse and a subsequent discharge activity lasting for >90 ms. The gamma-ray burst was recorded by both the high-sensitivity and low-sensitivity channels of the TGF detector, started at the beginning of the lightning flash and lasted for >90 ms.

**Fig. 4.**
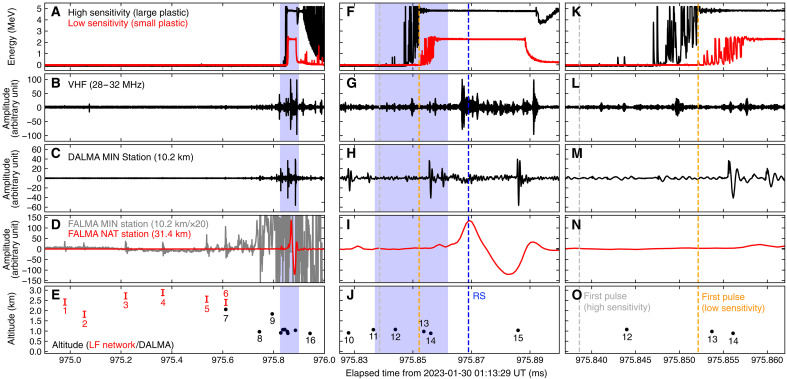
Waveforms of radiation and RF measurements. (**A** to **E**) waveforms before and during the TGF. (**F** to **J**) Expanded view of the waveforms during the TGF. (**K** to **O**) Further expanded view of the center panel. The displayed period is indicated by the blue region in the left and center panels. (A, F, and K) Waveforms of the high-sensitivity/large (black) and low-sensitivity/small (red) plastic scintillators onboard the TGF detector. (B), (G), and (L) Waveform of the VHF band (28 to 32 MHz) recorded by the TGF detector. (C), (H), and (M) Waveform of the HF band recorded by the MIN station of DALMA. (D), (I), and (N) Waveforms of the LF band recorded by MIN (gray) and NAT (red) stations of FALMA. The waveform of the MIN station is multiplied by 30 to display weak pulses before the RS and is not displayed in the right panel. (E), (J), and (O) Source altitudes of RF pulses estimated by the LF network (red) and DALMA (black). The numbers beside the plots correspond to those in Fig. 1. The location error of DALMA is almost the same as the marker size. The gray and orange dashed lines in the center and right panels show the first pulse of the downward TGF recorded by the high-sensitivity and low-sensitivity channels of the TGF detector, respectively.

The expanded figures of the waveforms are shown in Fig. 4. A return stroke (RS) was recorded by the LF network at 01:13:29.975869 UTC, with its peak current of −56 kA reported by the Japan Lightning Detection Network. Several LF pulses were detected leading to the RS, all of which have positive rising edges, i.e., the same polarity as negative cloud-to-ground discharge. The interval from the first LF pulse to the RS is 890 μs. Gamma rays are first detected by the high-sensitivity scintillator and then by the low-sensitivity scintillator. The high-sensitivity channel recorded the first photon at 01:13:29.975838 UTC, 31 μs before the RS, then detected multiple photons with energy of several MeV. Because of the pileup effect, the waveform becomes completely saturated 17 μs before the RS. The low-sensitivity channel recorded the first photons at 01:13:29.975852 UTC, 17 μs before the RS, and was saturated with pileup 12 μs before the RS. Saturation ended 23 and 19 μs after the RS for the high-sensitivity and low-sensitivity channels, respectively. During the gamma-ray burst, active RF emission was recorded in the VHF and the MF-HF bands.

At the beginning of the gamma-ray burst, the coincidence of high-sensitivity and low-sensitivity channels is not seen. This information is useful to distinguish TGFs from x-ray emissions from stepped leaders (*38*–*40*). The main difference between the two phenomena is photon energies; TGFs mainly consist of photons of megaelectron volts to tens of megaelectron volts, while x-ray emissions mainly consist of photons of below 1 MeV. The present gamma-ray burst includes many photons above 1 MeV detected by both the high-sensitivity and low-sensitivity channels, and it is apparently a TGF. On the other hand, we have to rule out the possibility of pileup. If multiple photons arrive to and react with a radiation detector simultaneously (typically within 100 ns with the present detector), then the detector only records the total energy deposit of those photons, rather than the energy of individual photons. Because x-ray emissions consist of multiple bunches of photons, whose duration is typically shorter than 1 μs, radiation detectors can experience pileups. In a situation where a pile-up occurs, multiple photons arrive at the detector simultaneously, and there is a high possibility that the photons will be measured simultaneously by multiple sensors. However, in the present case, there was no clear coincidence between the low-sensitivity and high-sensitivity channels before they were completely saturated, and hence, the MeV energy signals recorded by the TGF detector probably originate from the reaction of single photons, rather than a pileup. Therefore, it is concluded that the present case is not an x-ray emission from stepped leaders but a downward TGF.

The TGF detector recorded a burst of gamma rays for more than 80 ms after the TGF, especially in the large plastic scintillator (the high-sensitivity channel). This burst is thought to originate from photonuclear reactions, which is another evidence of a downward TGF (*11*, *41*–*43*). TGFs emit photons exceeding 10 MeV, and hence, photonuclear reactions such as N14+γ→13N+n and O16+γ→15O+n occur (*11*, *41*–*43*). Fast neutrons are generated by the reactions, and after being thermalized mainly by elastic scatterings with ^14^N, a neutron capture reaction N14+n→ ^15^N occurs. ^15^N produced by the neutron capture is in an excited state, so it immediately emits nuclear gamma rays and de-excites. A burst of the de-excited gamma rays is also called TGF afterglow (*44*). The duration of TGF afterglow depends on the timescale of the thermalization of neutrons by elastic scattering. At sea level, the time constant of thermalization is ∼50 ms, and the duration is several hundreds of milliseconds (*42*). Also, thermalized neutrons can be directly detected if they reach the ground before being captured by the atmospheric nuclei (*11*, *45*).

**Fig. 5.**
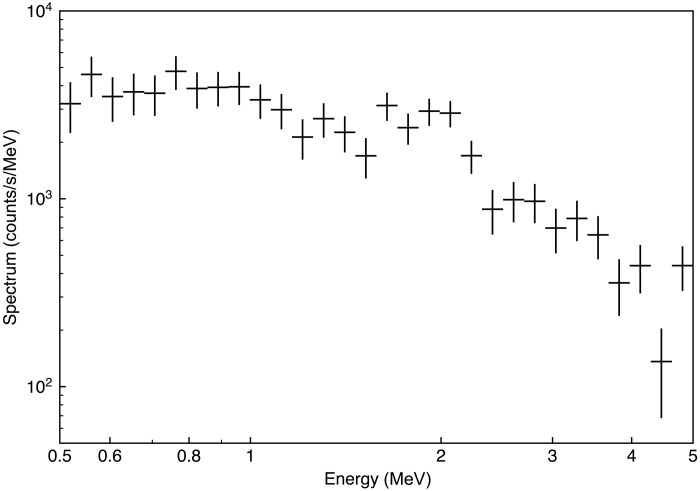
An energy spectrum of neutron origin after the downward TGF. The spectrum was obtained with the high-sensitivity channel of the TGF detector. The first 10 ms after the RS is excluded from the analysis to avoid the effect of saturation by the downward TGFs, and the total duration for the spectrum extraction is 86 ms, limited by the memory buffer of the TGF detector.

The energy spectrum of the burst recorded by the high-sensitivity channel is shown in Fig. 5. To avoid the effect of saturations by the downward TGF, the spectrum was extracted from 10 ms after the TGF onward. There is an edge-like feature in the spectrum, which corresponds to the Compton edge of 2.2 MeV photons emitted by the neutron capture reaction H1+n→2H+γ in the plastic scintillator, and is consistent with the result reported by Bowers *et al.* (*11*). A continuum component up to 5 MeV is detected, which is thought to originate from de-excitation gamma rays of 5.3 and 10.8 MeV from ^15^N.

The source positions of the RF pulses around the TGF detection time are located by the LF network and DALMA, as shown in Figs. 1 and 4. The IRI station of the LF network successfully recorded six LF pulses at the beginning of the lightning flash, but it was completely saturated afterward. Therefore, the source locations of the first six pulses are three-dimensionally determined by the LF network around the tower at altitudes ranging from 1.8 to 2.9 km. A minimum of 7 stations (pulse 2) and a maximum of 13 stations (pulse 6) were used for pulse locating. After the saturation of the IRI station, multiple DALMA stations recorded RF pulses, and their sources were three-dimensionally located by DALMA on the south side of Tower 1, mainly at an altitude of 0.8 to 0.9 km around the RS detection time. Also, the RS is located within 100 m of Tower 1 by 11 unsaturated stations of the LF network. The three stations far from Kanazawa—GFU, IBG, and KSM—are additionally excluded from the analysis.

**Fig. 6.**
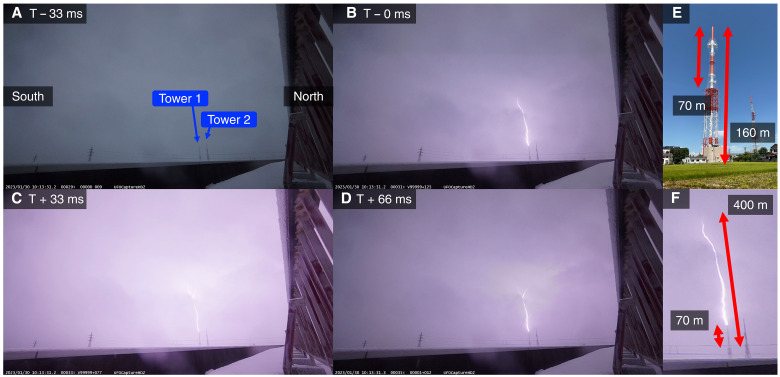
Optical images of the lightning discharge. Four panels show images 33 ms before (**A**), at the moment of (**B**), 33 ms after (**C**), and 66 ms after (**D**) the first frame of the lightning discharge. The left and right sides of the images correspond to the south and north, respectively. (**E**) and (**F**) provide scales of Tower 1 and the upward leader to estimate the cloud base.

The optical camera successfully recorded the lightning flash associated with the downward TGF, as shown in Fig. 6. A lightning strike can be seen on Tower 1 in the second frame (T − 0 ms). The lightning channel goes into clouds slightly south of Tower 1. In the third frame (T + 33 ms), the upward branching of the lightning channel can be seen just below the cloud, suggesting that an upward leader extended from Tower 1. The cloud base is estimated with the optical image and the height of Tower 1, as shown in Fig. 6. Tower 1 has a two-stage structure. The entire height is 160 m, and the height of the upper part is 70 m. In the optical image, the lower part of the tower is partially hidden, but by comparing the height of the upper part and the distance between the lowest point of the upper part and the cloud base, we estimated the cloud base to be ∼500 m. The broadcasting system of Tower 1 recorded a system anomaly for television broadcasting by the lightning discharge, which is another evidence of the lightning strike to Tower 1.

Photo credit: Yuuki Wada, Osaka University.

## DISCUSSION

The RS related to the TGF is considered to be a compact stroke (CS) (*15*, *46*, *47*). CS is an RS with a relatively high peak current and bipolar LF waveform, preceded by a downward negative leader with a duration of less than 1 ms. A downward activity is unclear in the three-dimensional location of the LF network. On the other hand, the LF pulses were located at higher than a 1.5-km altitude, and the polarity of the LF pulses is such that negative charges move downward. Therefore, a downward negative leader is considered to have preceded the CS. LF pulses coincident with TGFs, called slow pulse, have also been reported (*27*, *48*). The typical duration of slow pulses (50 to 100 μs) is similar to the present case, but it is not a slow pulse because slow pulses are usually associated with upward negative leader activities, not directly with RSs.

**Fig. 7.**
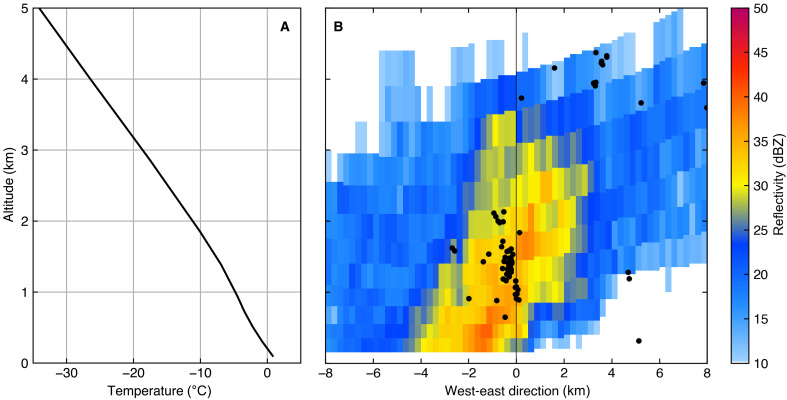
Vertical profiles of temperature and radar reflectivity. (**A**) Vertical profile of temperature at 36.6°N, 136.625°E, and 00:00 UT, 30 January 2023, obtained from the mesoscale model of Japan Meteorological Agency. (**B**) Vertical cross section of radar reflectivity for west-east direction observed by the Noumi X-band radar. The black line in the right panel shows the position of Tower 1. The black dots in the right panel are MF-HF pulses located by DALMA within 1 km from the west-east line passing Tower 1.

The existence of the downward negative leader is also supported by a radar observation. Figure 7 shows a vertical profile of temperature and a west-east vertical cross section of radar reflectivity. The temperature profile is extracted from initial analysis data of the mesoscale model produced by the Japan Meteorological Agency, and the radar profile is composited from multiple plan-position indicator scans within 5 min by the Nomi X-band weather radar of the eXtended RAdar Information Network (XRAIN). Lightning pulses located by DALMA within 1 km in the north-south direction from the east-west cross section are also shown by black dots.

Radar echoes exceeding 35 dBZ were observed below 3 km, suggesting the presence of graupels. DALMA also located many lightning pulses at around 1- to 2-km altitudes during the lightning flash associated with the TGF. The vertical temperature profile shows the −10°C altitude of 1.8 km, where negative charges are generally accumulated (*49*, *50*). Therefore, a negative charge layer was formed around an altitude of 2 km, and hence, a negative downward leader can be initiated in this region.

The optical camera confirmed that a cloud-to-ground strike occurred on Tower 1 during the lightning flash. While the time resolution of the optical camera (33 ms) does not allow direct determination of the progression direction of leaders, the captured image shows an upward leader branch, which allows us to conclude an upward leader originating from Tower 1. This upward leader is inferred to be a positive leader by a smooth leader trajectory and fewer branches than typical negative leaders. With the limited time resolution, it is difficult to investigate whether the cloud-to-ground strike to Tower 1 is associated with the TGF by only the optical camera. On the other hand, the LF network localized the RS associated with the TGF within 100 m of Tower 1, and hence, it is reasonable to consider that the discharge path shown in the first frame corresponds to the RS. By combining the observation results of the optical camera and the LF network, it is concluded that an upward positive leader extended from Tower 1 around the occurrence time of the downward TGF.

At the moment of the TGF detection and the CS, DALMA located MF-HF pulses at an altitude of 0.8 to 0.9 km. By combining the observations, we get a picture leading to the CS:

1) A downward negative leader initiated 890 μs before the CS, at an altitude of 2 to 3 km, was extending toward the ground.

2) An upward positive leader initiated and was extending upward from Tower 1, although the initiation timing was not clear.

3) The downward negative and upward positive leaders were approaching each other, and they collided at an altitude of 0.8 to 0.9 km (attachment process), which resulted in the CS.

The attachment process seems to have occurred in the cloud, given that the cloud base is estimated to be ∼0.5 km.

**Fig. 8.**
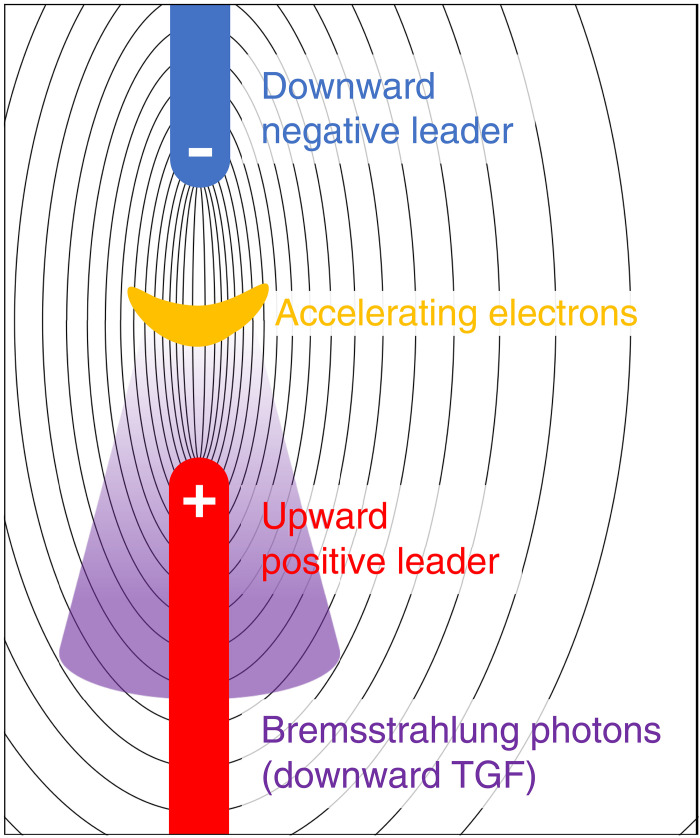
A schematic of two colliding leaders producing the TGF. The black lines show electrical flux lines produced by the two leaders.

The downward TGF started at least 31 μs before the CS. This suggests that the TGF occurred when the two leaders were approaching and just before they collided. A possible scenario to trigger the TGF is that an electric field concentrates between the two leaders as they approach each other, accelerating and multiplying high-energy electrons. Figure 8 shows a conceptual diagram of this scenario. The electron acceleration/amplification mechanism in TGF is not well understood, and the relativistic feedback model (*19*, *20*, *51*) and the thermal runaway model (*21*, *22*, *52*, *53*) have been proposed so far. While it remains unclear which mechanism worked in the present case, this scenario suggests that the intensified electric field in a relatively compact area between the leaders facilitated the production of high-energy electrons. In particular, discussions on electron acceleration in a leader tip may be applied to the present scenario (*51*, *53*).

The average speed of the downward negative leader is estimated to be 1.8 × 10^6^ m/s as it initiated at an altitude of ∼2.5 km and 890 μs before the CS. Assuming that the upward positive leader initiated at the same time as the negative one, the leader speed is also estimated to be 0.9 × 10^6^ m/s. This speed of the downward negative leader is faster than a typical case in winter lightning but is common in CSs (*36*, *46*, *47*) and is suggestive of higher electrical potential than typical winter lightning.

Recent observations by high-speed cameras have much improved the understanding of the attachment process (*54*–*57*). In the case of typical negative cloud-to-ground discharge, a negative downward leader approaches the ground, and then one or multiple upward positive leaders, referred to as upward connecting leaders, extend from the ground. When the two leaders in opposite polarities approach, a common streamer zone is formed between the two leaders, and the attachment process initiates. The length of upward connecting leaders is typically tens of meters in the case of natural negative discharges in summer (*55*–*57*). There are also several reported cases with upward connecting leaders extending 100 to 200 m (*54*, *58*), longer than the typical length. On the other hand, our observation suggests that the upward leader extended ∼800 m from the tip of Tower 1, exceptionally longer than the previously reported cases. This also indicates that the present case involved an electric field between two leaders higher than that of typical negative cloud-to-ground discharge. Also, previous observations indicate that a common streamer zone starts to form typically a few microseconds before RS. However, the first photon of the TGF was detected by the high-sensitivity channel at least 31 μs before the CS, and hence, the TGF is thought to have initiated before a common streamer zone formed.

The proposed scenario suggests that the intensified electric field decays, and a TGF ends immediately after the RS. On the other hand, the saturation of the TGF detector continued for ∼20 μs after the CS, contradicting the scenario. We have to consider three points for the saturation after the CS. First is the saturation behavior of the TGF detector. To verify the situation, we prepared a spare electric circuit and photomultiplier, and an light-emitting diode (LED) was used to imitate saturating scintillation lights from the plastic scintillators. In an extreme condition, the saturation of the electric circuit output continues for more than 10 μs after the LED turns off. Therefore, it is possible that the TGF ended >10 μs before the saturation ended. Second is the propagation time of photons. Because the TGF detector was installed near Tower 1 and the attachment process was estimated to occur at an altitude of 0.8 to 0.9 km, photons reached the TGF detector with a delay of at least ∼3 μs. A portion of photons undertook multiple scatterings in the atmosphere and could have had an additional delay of a few microseconds. These two factors may be enough to explain the ∼20 μs saturation after the CS. The third possibility is that the acceleration and multiplication of electrons continued even after the CS. If the acceleration and multiplication continued after the current peak of the CS, then a mechanism to maintain electric fields in the lightning path may have existed, and further modeling is needed.

In previous studies, the relation between negative leaders, RSs, and TGFs was discussed using RF observations (*26*, *31*). On the other hand, RF emissions from positive leaders are generally weak, making them difficult to detect with LF and VHF bands, and hence, optical observation is necessary (*9*–*11*). In the present case, the simultaneous observation of optical and RF bands suggests the existence of both upward positive and downward negative leaders. It is possible that upward positive leaders associated with TGFs and/or RSs were missed in RF measurements so far (*29*).

Wada *et al.* (*13*) classified LF pulses associated with downward TGFs in winter thunderstorms into two types: energetic-bipolar and small-bipolar types. TGFs of the energetic-bipolar type are coincident with high–peak current LF pulses (>100 kA), while those of the small-bipolar type are followed by bipolar LF pulses of tens of kiloamperes. The present case can be categorized into the small-bipolar type because the peak current of the RS is not as large as −100 kA, and the TGF initiated before the RS. In the cases of the small-bipolar type presented in (*13*), TGFs preceded RSs and also followed positive-onset LF pulses, indicative of downward negative leaders. While upward positive leaders were not confirmed by Wada *et al.* (*13*) due to limited observation facilities, the other characteristics of the small-bipolar type are consistent with the present case.

Moore *et al.* (*38*) and Dwyer *et al.* (*39*) reported high-energy emissions related to RSs. They detected x-ray emission from leaders and possibly from RSs. X-ray emission from leaders is recognized as a different phenomenon from TGFs. On the other hand, x-ray emission coincident with RSs, particularly the cases of Dwyer *et al.* (*39*), could have taken place in a similar situation as the present case. The current understanding of x-ray emission is that the tip of leaders produces electrons of several hundreds of kiloelectron volts by the thermal runaway process. If there is a large potential, these sub–megaelectron volt electrons can be accelerated to tens of megaelecton volts. As a possibility, thermal runaway electrons could have been produced in a similar context to x-ray emission and accelerated up to tens of megaelectron volts by a stronger electric field to cause the downward TGF.

In conclusion, we performed a multifrequency observation campaign aiming at winter thunderstorms with high energy, optical, and RF instruments and successfully detected a downward TGF coincident with a cloud-to-ground discharge to a television transmission tower. A downward negative leader, identified by the LF network, initiated 890 μs before an RS at ∼2.5-km altitude. A positive upward leader was also extending from a television transmission tower, confirmed by the optical camera. Two leaders collided at an altitude of 0.8 to 0.9 km, confirmed by DALMA, and the RS of −56 kA occurred. The length of the positive upward leader is exceptionally longer than typical upward connecting leaders of negative cloud-to-ground discharge. The RS can be classified as CS, which has a relatively high–peak current and bipolar LF waveform, preceded by a downward negative leader with a duration of less than 1 ms. Because the onset of the TGF was detected 31 μs before the CS, the electron acceleration and multiplication responsible for the TGF is inferred to have been initiated by a strong and compact electric-field region between the two leaders in opposite polarities.
